# Supplementary material for: Motor dysfunction and neurodegeneration in a C9orf72 mouse line expressing poly-PR
Source: Nat Commun. 2019 Jul 2;10:2906. doi: 10.1038/s41467-019-10956-w (PMC6606620; doi:10.1038/s41467-019-10956-w)
Supplement: Supplementary file 1 — Supplementary Information [file 41467_2019_10956_MOESM1_ESM.pdf]

SUPPLEMENTARY INFROMATION

Motor dysfunction and neurodegeneration in a *C9orf72* mouse line expressing poly-PR

Hao et al

Supplementary Figure 1

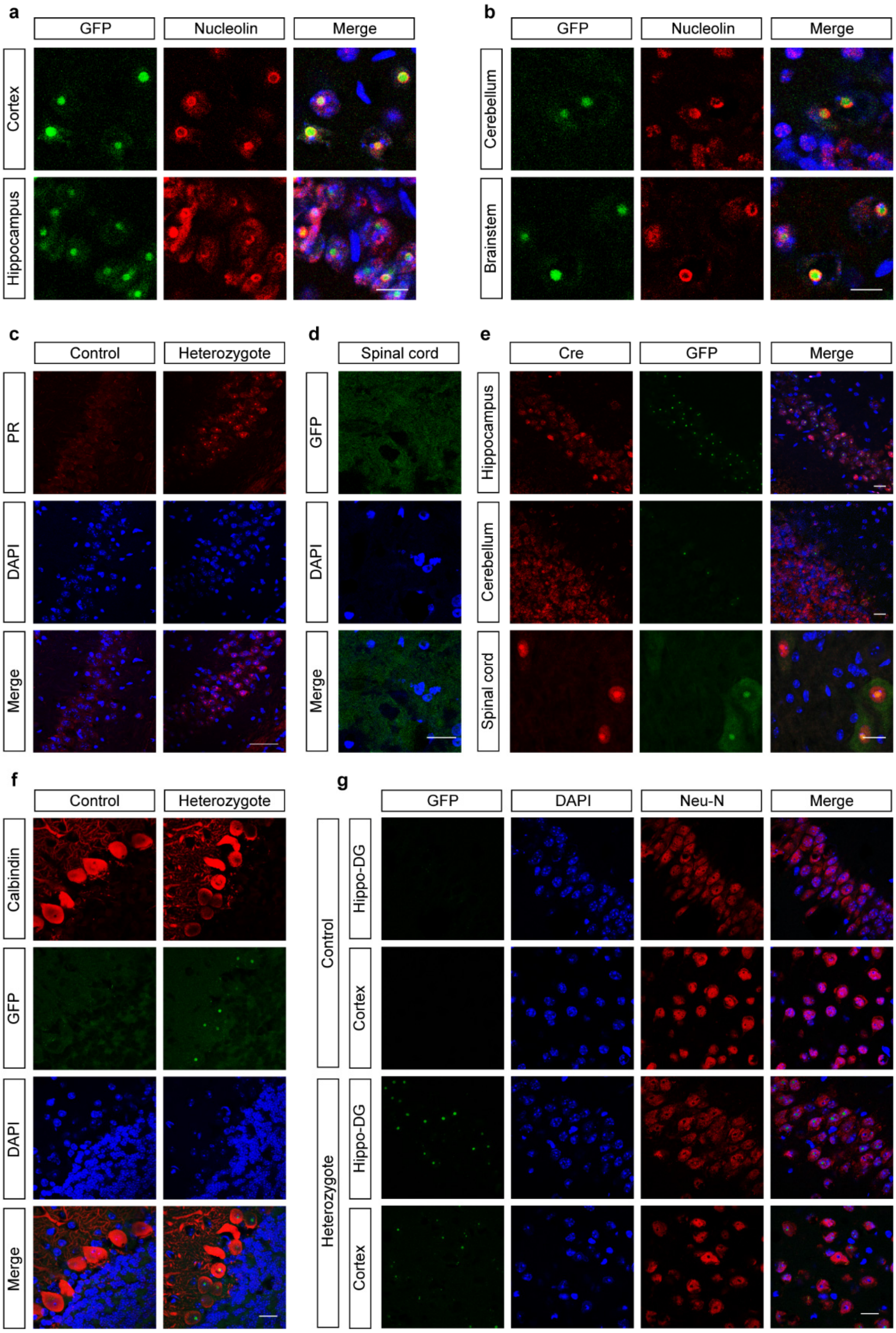

Supplementary Fig. 1 | Distribution of GFP-PR$_{28}$ in brain regions of heterozygous mice. **a, b** Representative images show co-localization of GFP-PR$_{28}$ and nucleolin in cortex, hippocampus, cerebellum and brainstem of 2 months old control and GFP-PR$_{28}$ heterozygous mice. Scale bar represents 50 µm. **c** Distribution of GFP-PR$_{28}$ in hippocampal neurons of 2

months old control and GFP-PR_{28} heterozygous mice validated with PR antibody. Scale bar represents 50 µm. **d** Representative images showing distribution of GFP staining in lumbar spinal cord of control mice at 2 months of age. Scale bar represents 50 µm. **e** Distribution of GFP-PR_{28} and Cre recombinase in hippocampus, cerebellum and spinal cord of 2 months old GFP-PR_{28} heterozygous mice, Scale bar represents 20 µm. **f** Distribution of GFP-PR_{28} in calbindin positive Purkinje cells in the cerebellum of 2 months old control and GFP-PR_{28} heterozygous mice. Scale bar represents 20 µm. **g** Distribution of GFP-PR_{28} in Neu-N positive neurons in the hippocampus and motor cortex of 2 months old control and GFP-PR_{28} heterozygous mice. Scale bar represents 20 µm.

Supplementary Figure 2

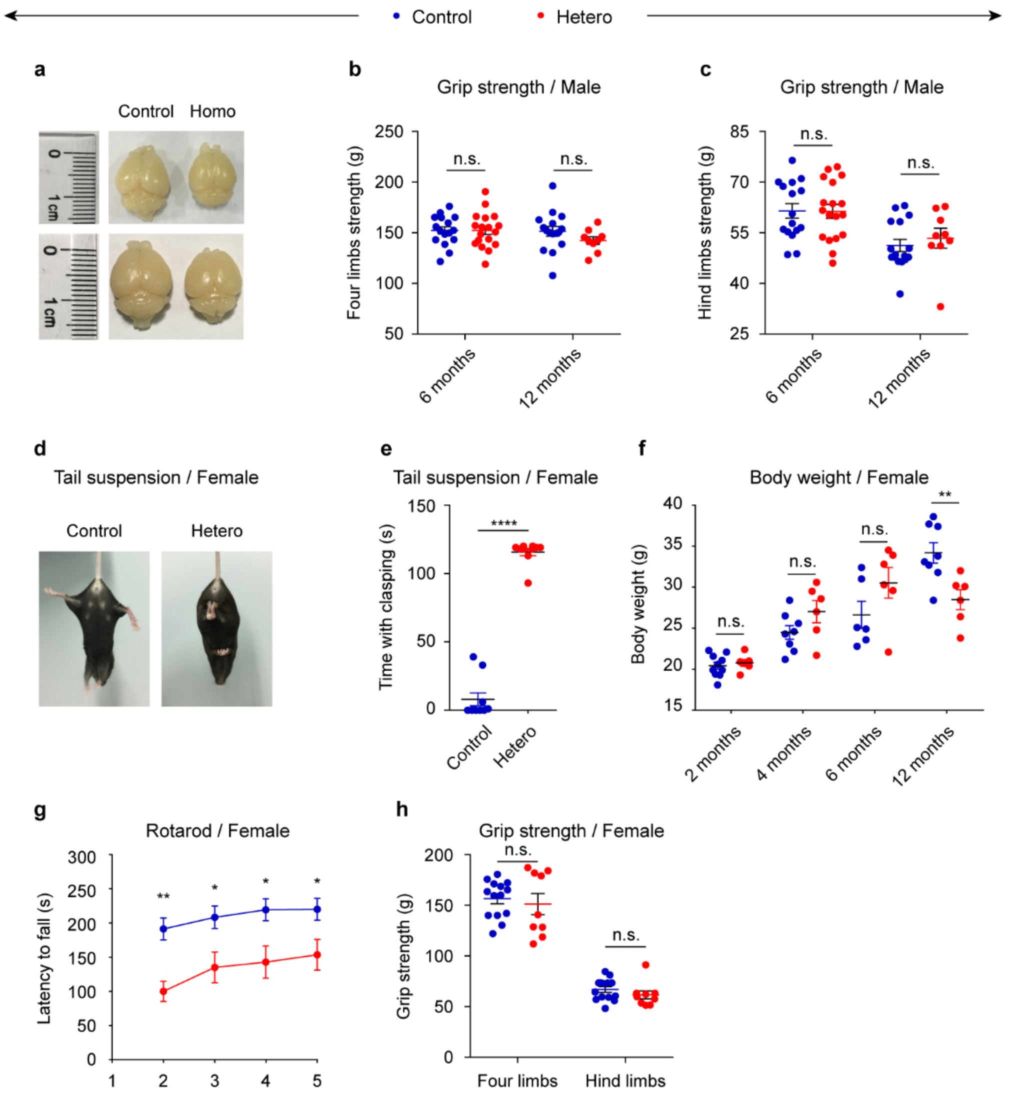

Supplementary Fig. 2 | GFP-PR$_{28}$ transgenic female mice show motor deficits.

a Representative images showing brain size of 20 days old control and the homozygous mice. **b** Grip strength test of four limbs of 6 months old male control and GFP-PR$_{28}$ heterozygous mice. Two-way ANOVA, Bonferroni post hoc test; 6 months, n = 16, 18 mice; 12 months, n = 16, 9 mice. **c** Grip strength test of hind limbs of 6 months old male control and GFP-PR$_{28}$ heterozygous mice. Two-way ANOVA, Bonferroni post hoc test; 6 months, n = 16, 18 mice; 12 months, n = 16, 9 mice. **d** Representative images of female control and GFP-PR$_{28}$ heterozygous mice at 2 months of age in tail suspension test. **e** Quantification of the clasping time of mice in (**d**) during 2 min test. Mann Whitney test, n = 10, 10 mice. **f** Body weight of female control and GFP-PR$_{28}$ heterozygous mice at 2, 4, 6 and 12 months of age. Two-way ANOVA, Bonferroni post hoc test; 2 months, n = 10, 6 mice; 4 months, n = 8, 6 mice; 6 months, n = 6, 6 mice; 12 months, n = 8, 6 mice. **g** Latencies to fall from the accelerated rotating beams of female control and GFP-PR$_{28}$ heterozygous mice at 6 months of age. Two-way ANOVA, Bonferroni post hoc

test; *n* = 18, 13 mice. **h** Grip strength test of four limbs and hind limbs of female control and GFP-PR_{28} heterozygous mice at 6 months of age. Two-way ANOVA, Bonferroni post hoc test; four limbs, *n* = 14, 9 mice; hind limbs, *n* = 14, 9 mice. All data were displayed as mean ± s.e.m. $*P < 0.05$, $**P < 0.01$, $****P < 0.0001$, n.s., no significant.

Supplementary Figure 3

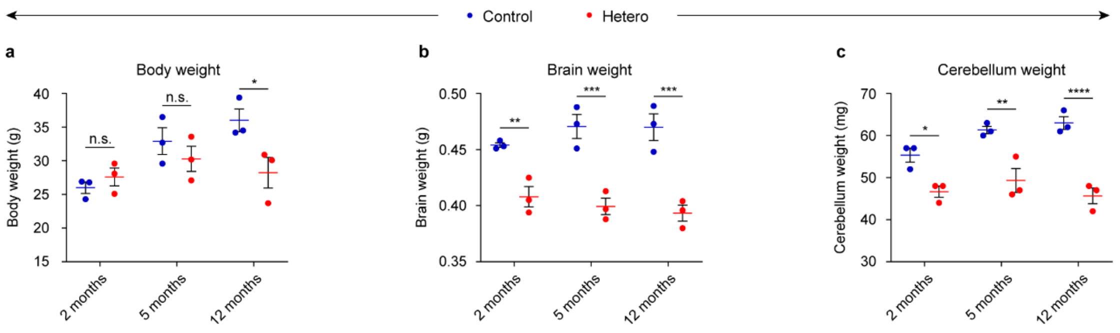

Supplementary Fig. 3 | The expression of GFP-PR$_{28}$ causes brain atrophy.

a-c Quantification of body weight (**a**), brain weight (**b**) and cerebellum weight (**c**) of control and GFP-PR$_{28}$ heterozygous mice at 2, 5 and 12 months of age. $n = 3$, 3 mice. All data were displayed as mean ± s.e.m. Two ANOVA, Bonferroni post hoc test; *$P < 0.05$, **$P < 0.01$, ***$P < 0.001$, ****$P < 0.0001$, n.s., no significant.

Supplementary Figure 4

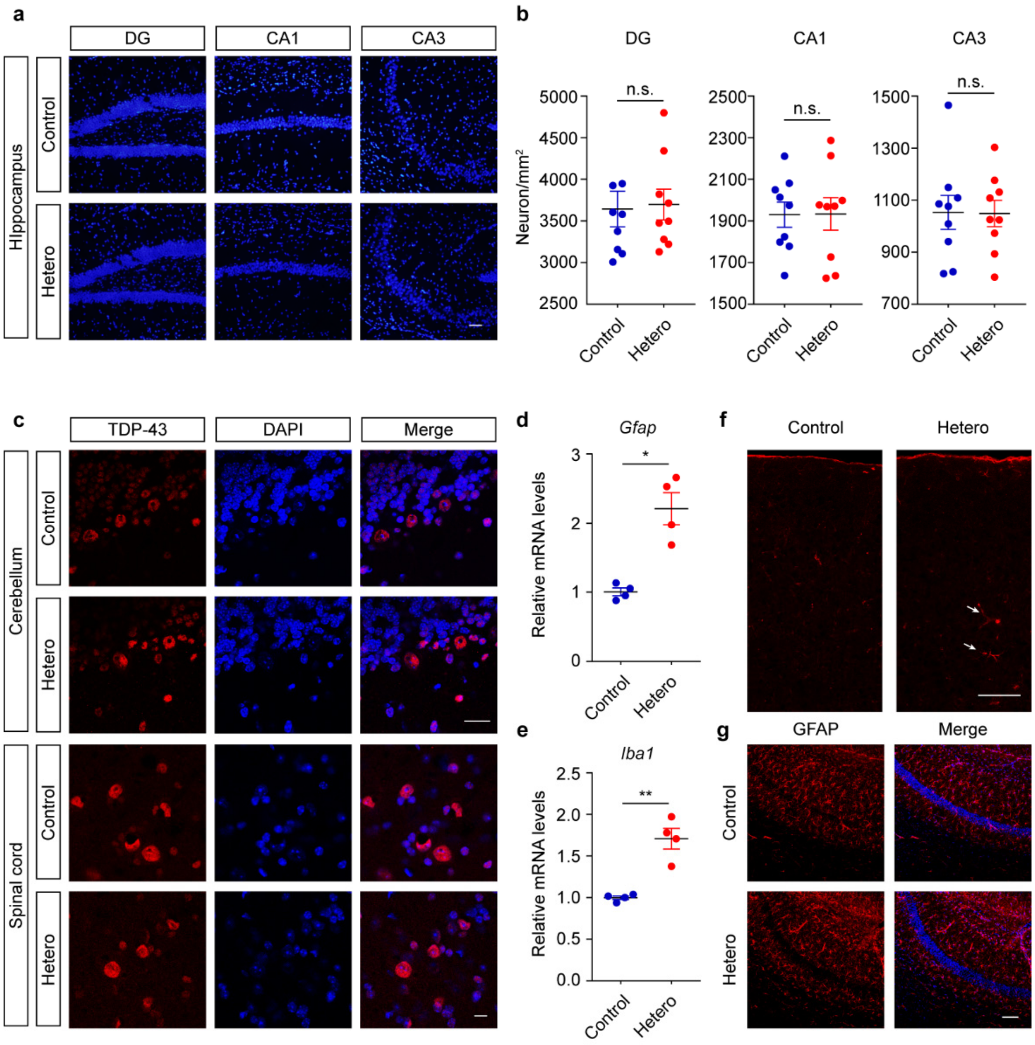

Supplementary Fig. 4 | GFP-PR$_{28}$ heterozygous mice show no hippocampal neuronal deficits.

a Representative images showing the numbers of Hoechst positive neurons in the hippocampus of 6 months old control and GFP-PR$_{28}$ heterozygous mice. Dentate gyrus (DG), cornu ammonis 1 (CA1), cornu ammonis 3 (CA3). Hoechst (blue). Scale bar represents 100 µm. **b** Quantification of cell numbers in different hippocampal regions in (**a**). Two-tailed *t* test, *n* = 9, 9 mice. **c** Representative images showing the localization of TDP-43 in the cerebellum and spinal cord of 12 months old control and GFP-PR$_{28}$ heterozygous mice. Scale bar represents 20 µm. **d** Relative mRNA levels of *Gfap* in the cerebellum 6 months old control and GFP-PR$_{28}$ heterozygous mice determined by quantitative real-time PCR. Two-tailed *t* test, *n* = 4, 4 mice. **e** Relative mRNA levels of *Iba1* in the cerebellum 6 months old control and GFP-PR$_{28}$ heterozygous mice determined by quantitative real-time PCR. Two-tailed *t* test, *n* = 4, 4 mice. **f** Representative images showing the staining of GFAP positive astrocytes in the motor cortex

of 6 months old control and GFP-PR_{28} heterozygous mice. Arrows indicate the activation of astrocytes. Scale bar represents 100 μm. **g** Representative images showing the staining of GFAP positive astrocytes in the hippocampus of 6 months old control and GFP-PR_{28} heterozygous mice. Scale bar represents 100 μm. All data were displayed as mean ± s.e.m. $*P < 0.05$, $**P < 0.01$, n.s., no significant.

Supplementary Figure 5

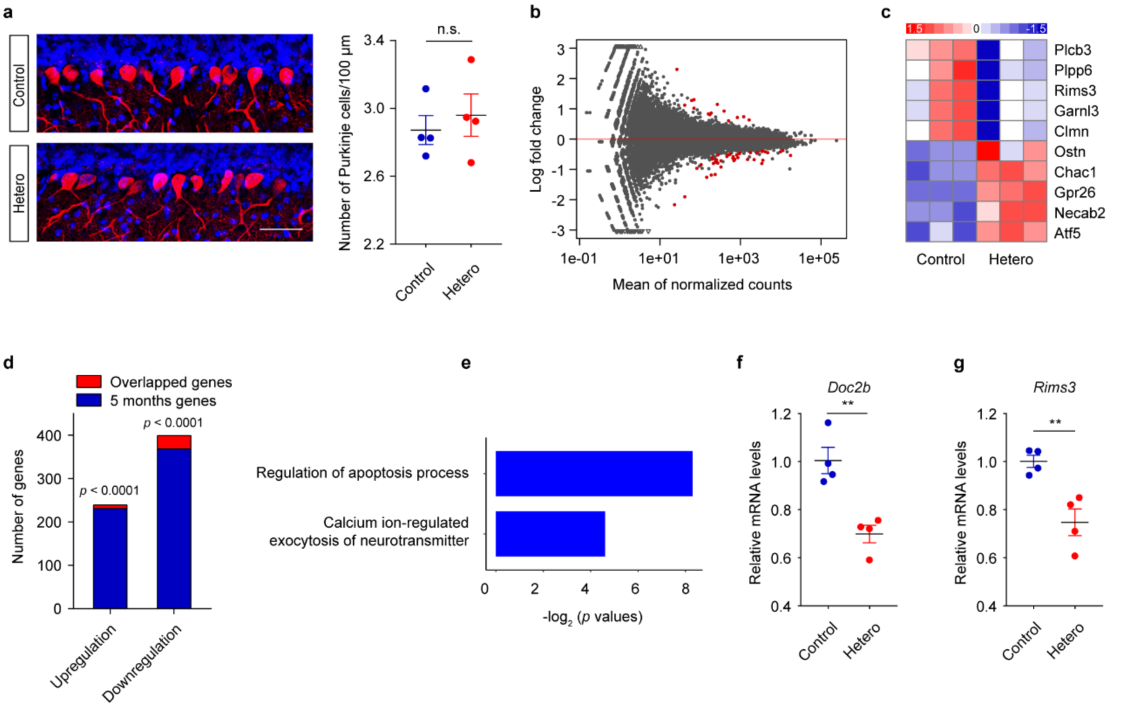

Supplementary Fig. 5 | Dysregulated synaptic-related genes in the cerebellum of 2 months old mice.

a Left, representative images showing the numbers of Purkinje cells in 2 months old control and GFP-PR_{28} heterozygous mice. Calbindin (red), Hoechst (blue). Scale bar represents 100 μm. Right, quantification of the numbers of calbindin positive Purkinje cells from (Left). Two-tailed *t* test, $n = 4, 4$ mice. **b** MA-plot of differentially expressed genes in the cerebellum of 2 months old control and GFP-PR_{28} heterozygous mice. Red blots indicate significant changes, $n = 3, 3$ mice. **c** Hierarchical clustering of top ten differentially expressed genes in the cerebellum of 2 months old control and GFP-PR_{28} heterozygous mice. **d** Comparison of differentially expressed genes in the cerebellum of 2 and 5 months old heterozygous mice. 2 months old, 11 upregulated genes, 8 overlapped genes; 31 downregulated genes, 30 overlapped genes. **e** Gene Ontology (GO) biological processes analyses of upregulated and downregulated genes in (**b**). No significant pathway indicated by upregulated genes of 2 months old heterozygous mice. **f, g** Relative mRNA levels of *Doc2b* and *Rims3* in the cerebellum of 2 months old control and GFP-PR_{28} heterozygous mice determined by qRT-PCR. Two-tailed *t* test, $n = 4, 4$ mice. All data were displayed as mean ± s.e.m. $^{**}P < 0.01$, n.s., no significant.

Supplementary Figure 6

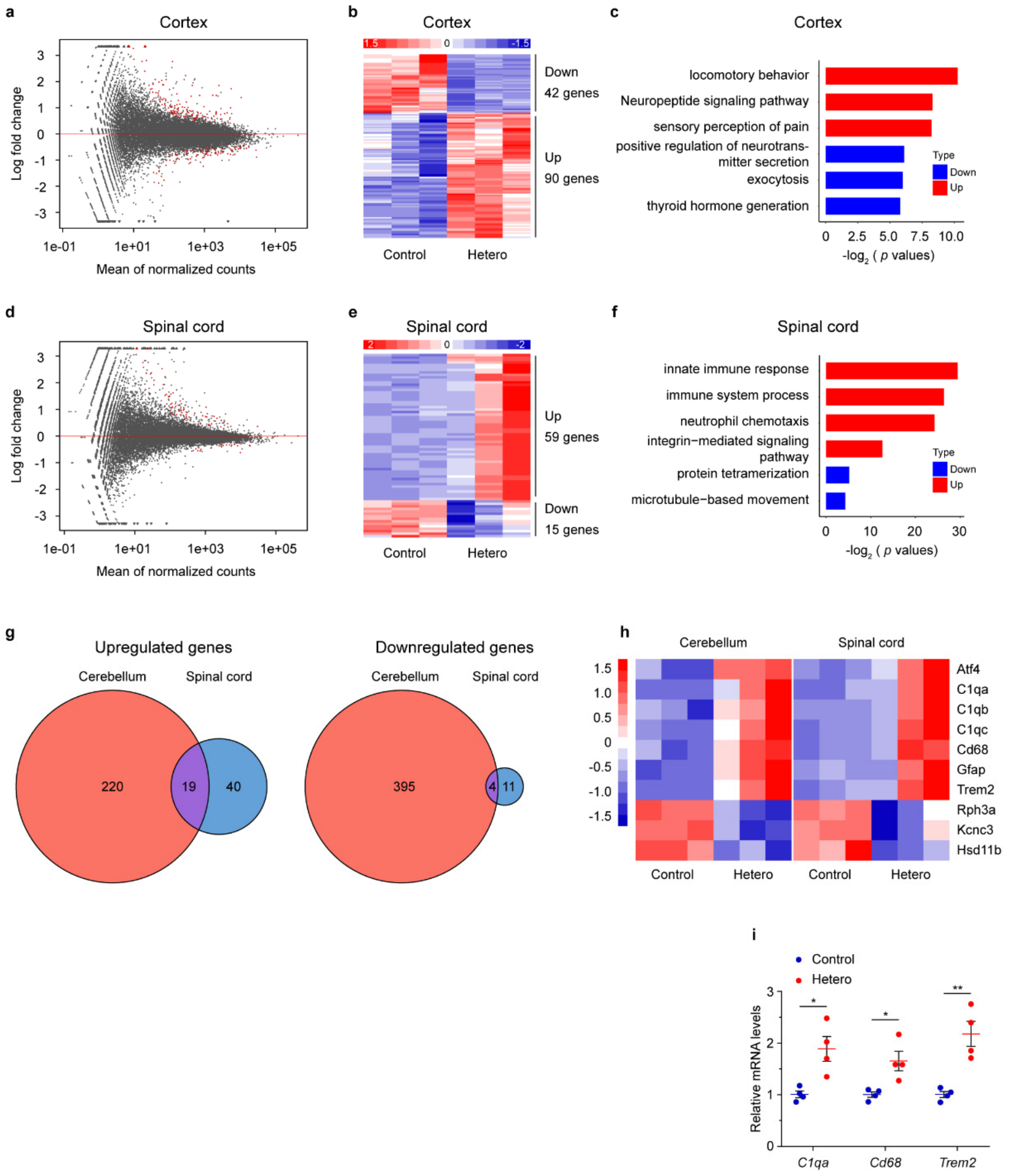

Supplementary Fig. 6 | RNA-seq analyses in the cortex and lumbar spinal cord of 6 months old mice.

a MA-plot of differentially expressed genes in the cortex of 6 months old control and GFP-PR$_{28}$ heterozygous mice. Red blots indicate significant changes, *n* = 3, 3 mice. **b** Hierarchical clustering of differentially expressed genes in the cortex of 6 months old control and GFP-PR$_{28}$ heterozygous mice. **c** Gene Ontology (GO) biological processes analyses of upregulated and downregulated genes in (**b**). **d** MA-plot of differentially expressed genes in the lumbar spinal cord of 6 months old control and GFP-PR$_{28}$ heterozygous mice. Red blots indicate significant changes, n = 3, 3 mice. **e** Hierarchical clustering of differentially expressed genes in the lumbar spinal cord of 6 months old control and GFP-PR$_{28}$ heterozygous mice. **f** Gene Ontology (GO)

biological processes analyses of upregulated and downregulated genes in (**e**). **g** Comparison of differentially expressed genes in the cerebellum and spinal cord of heterozygous mice. **h** Representative ten genes both dysregulated in the cerebellum and lumbar spinal cord of heterozygous mice. **i** Representative genes dysregulated in (**h**) were determined by qRT-PCR. Two-tailed *t* test, $n = 4, 4$ mice. All data were displayed as mean ± s.e.m. $^{*}P < 0.05$, $^{**}P < 0.01$.

Supplementary Table 1 | Primers used for transgenic mice genotyping.

Target	Sense/Antisense	Sequence
F1	Forward	5'-AGTCGCTCTGAGTTGTTATCAG-3'
R1	Reverse	5'-TGAGCATGTCTTTAATCTACCTCGATG-3'
F2	Forward	5'-GCATCGATACCGTCGACCTC-3'
R2	Reverse	5'-TTTGATAAGGCTGCAGAAGGAGCGG-3'
Thy1-cre	Forward	5'-GCGGTCTGGCAGTAAAAACTATC-3'
Thy1-cre	Reverse	5'-GTGAAACAGCATTGCTGTCACTT-3'

Supplementary Table 2 | Primers used for quantitative real time PCR.

Target	Forward	Reverse
Gfp	5'-GAAGCGCGATCACATGGT-3'	5'-CCATGCCGAGAGTGATCC-3'
Gfap	5'-GGAGAGGGACAACTTTGCAC-3'	5'-AGCCTCAGGTTGGTTTCATC-3'
Iba1l	5'-GGATTTGCAGGGAGGAAAAG-3'	5'-TGGGATCATCGAGGAATTG-3'
β-actin	5'-GGCTGTATTCCCCTCCATCG-3'	5'-CCAGTTGGTAACAATGCCATGT-3'
Camk4	5'-GAGAACCTCGTCCCGGATTAC-3'	5'-ACACAATGGATGTAGCACCCC-3'
Grin2a	5'-ACGTGACAGAACGCGAACTT-3'	5'-TCAGTGCGGTTCATCAATAACG-3'
Kcnj9	5'-ACCGCCTCTTTCTCGTCTCA-3'	5'-GATCTCGAAGTCGTCCCTCTC-3'
Rims3	5'-GGAATGTAGTAAGGAGTTCCAGC-3'	5'-CTGGGTCAAGCCGACGATAG-3'
Syt2	5'-AGAACCTGGGCAAATTGCAGT-3'	5'-CCTAACTCCTGGTATGGCACC-3'
Unc13a	5'-GCTGTGCGTGGGAGTCAAA-3'	5'-CAGCTATGGTAGTGCTCTTCAC-3'
Pcp2	5'-ACAGTTAATTCCCTGCCTGG-3'	5'-CTCAAGGAGCTTGTGTCTGG-3'
Doc2b	5'-CGACGGCTACGAGTCAGAC-3'	5'-TTCAGGGTGTTCCGAAGAGTT-3'
C1qa	5'-AAAGGCAATCCAGGCAATATCA-3'	5'-TGGTTCTGGTATGGACTCTCC-3'
Cd68	5'-TGTCTGATCTTGCTAGGACCG-3'	5'-GAGAGTAACGGCCTTTTTGTGA-3'
Trem2	5'-CTGGAACCGTCACCATCACTC-3'	5'-CGAAACTCGATGACTCCTCGG-3'
