# Supplementary material for: Motor dysfunction and neurodegeneration in a C9orf72 mouse line expressing poly-PR
Source: Nat Commun. 2019 Jul 2;10:2906. doi: 10.1038/s41467-019-10956-w (PMC6606620; doi:10.1038/s41467-019-10956-w)
Supplement: Supplementary file 3 — Description of Additional Supplementary Files [file 41467_2019_10956_MOESM3_ESM.pdf]

Description of Additional Supplementary Files

File Name: Supplementary Data 1
Description: Differentially expressed genes of the cerebellum at 5 months old.

File Name: Supplementary Data 2
Description: Differentially expressed genes of the cerebellum at 2 months old.

File Name: Supplementary Data 3
Description: Differentially expressed genes of the cortex at 6 months old.

File Name: Supplementary Data 4
Description: Differentially expressed genes of the spinal cord at 6 months old.

File Name: Supplementary Movie 1
Description: Movie of the 6 months old control mice walking along the edges of cage.

File Name: Supplementary Movie 2
Description: Movie of the 6 months old heterozygous mice walking along the edges of cage.
