# Supplementary material for: Motor dysfunction and neurodegeneration in a C9orf72 mouse line expressing poly-PR
Source: Nat Commun. 2019 Jul 2;10:2906. doi: 10.1038/s41467-019-10956-w (PMC6606620; doi:10.1038/s41467-019-10956-w)
Supplement: Supplementary file 10 — Reporting Summary [file 41467_2019_10956_MOESM10_ESM.pdf]

nature research

Corresponding author(s): Guanghui Wang

Last updated by author(s): Jun 3, 2019

Reporting Summary

Nature Research wishes to improve the reproducibility of the work that we publish. This form provides structure for consistency and transparency in reporting. For further information on Nature Research policies, see Authors & Referees and the Editorial Policy Checklist.

Statistics

For all statistical analyses, confirm that the following items are present in the figure legend, table legend, main text, or Methods section.

n/a	Confirmed	
☐	☒	The exact sample size (*n*) for each experimental group/condition, given as a discrete number and unit of measurement
☐	☒	A statement on whether measurements were taken from distinct samples or whether the same sample was measured repeatedly
☐	☒	The statistical test(s) used AND whether they are one- or two-sided<br>*Only common tests should be described solely by name; describe more complex techniques in the Methods section.*
☒	☐	A description of all covariates tested
☐	☒	A description of any assumptions or corrections, such as tests of normality and adjustment for multiple comparisons
☐	☒	A full description of the statistical parameters including central tendency (e.g. means) or other basic estimates (e.g. regression coefficient) AND variation (e.g. standard deviation) or associated estimates of uncertainty (e.g. confidence intervals)
☐	☒	For null hypothesis testing, the test statistic (e.g. *F*, *t*, *r*) with confidence intervals, effect sizes, degrees of freedom and *P* value noted<br>*Give P values as exact values whenever suitable.*
☒	☐	For Bayesian analysis, information on the choice of priors and Markov chain Monte Carlo settings
☒	☐	For hierarchical and complex designs, identification of the appropriate level for tests and full reporting of outcomes
☒	☐	Estimates of effect sizes (e.g. Cohen's *d*, Pearson's *r*), indicating how they were calculated

Our web collection on statistics for biologists contains articles on many of the points above.

Software and code

Policy information about availability of computer code

Data collection	No software was used to collect data.
Data analysis	Trimmomatic (version 0.30)<br>Hisat2 (version 2.0.1)<br>HTSeq (version 0.6.1)<br>R (version 3.4.4)<br>RStudio (version 1.1442)<br>ggplot2 (version 2.21)<br>DESeq2 (version 1.18.1)<br>pheatmap (version 1.0.10)<br>VennDiagram (version 1.6.20)<br>CellSense (version standard)<br>Zen blue edition(version 2012)<br>Fiji with Image J (1.51n)<br>GraphPad Prism (version 7.00)<br>No specific software and code was used in the study.

For manuscripts utilizing custom algorithms or software that are central to the research but not yet described in published literature, software must be made available to editors/reviewers. We strongly encourage code deposition in a community repository (e.g. GitHub). See the Nature Research guidelines for submitting code & software for further information.

Data

Policy information about availability of data

All manuscripts must include a data availability statement. This statement should provide the following information, where applicable:

- Accession codes, unique identifiers, or web links for publicly available datasets
- A list of figures that have associated raw data
- A description of any restrictions on data availability

The data of figs. 1e, 1g, 2b, 2c, 3b, 3c, 3d, 3e, 3g, 3h, 3i, 4a, 4b, 4c, 4d, 4e, 5b, 5d, 5f, 5h, 6c, 6d, 6f, 7e, 7f, and Supplementary figs. 2b, 2c, 2e, 2f, 2g, 2h, 3a, 3b, 3c, 4b, 4d, 4e, 5a, 5f, 5g, 6i are provided as a Source Data file.
The authors will make all data available to readers upon reasonable request.
The RNA sequencing data of this article have been deposited in the NCBI GEO database under accession number GSE132108.

Field-specific reporting

Please select the one below that is the best fit for your research. If you are not sure, read the appropriate sections before making your selection.

☒ Life sciences ☐ Behavioural & social sciences ☐ Ecological, evolutionary & environmental sciences

For a reference copy of the document with all sections, see nature.com/documents/nr-reporting-summary-flat.pdf

Life sciences study design

All studies must disclose on these points even when the disclosure is negative.

Sample size	For behavioral tests, at least 5 mice in each group were used,for qRT-PCR. For Western blotting and RNA sequencing experiments, at least 3 mice in each group were used, this allows us to find statistically significant results. Sample sizes are shown in respective figure legend.
Data exclusions	No data were excluded from the results.
Replication	All attempts at replication were successful.
Randomization	Mice genotyping were performed at 20 days of age, the mice from behavioral tests and RNA sequencing experiments were randomly selected from each group.
Blinding	Behavioral test and quantification of neuropathology was performed by investigators blinded to experimental groups.

Reporting for specific materials, systems and methods

We require information from authors about some types of materials, experimental systems and methods used in many studies. Here, indicate whether each material, system or method listed is relevant to your study. If you are not sure if a list item applies to your research, read the appropriate section before selecting a response.

Materials & experimental systems

n/a	Involved in the study
☐	☒ Antibodies
☒	☐ Eukaryotic cell lines
☒	☐ Palaeontology
☐	☒ Animals and other organisms
☒	☐ Human research participants
☒	☐ Clinical data

Methods

n/a	Involved in the study
☒	☐ ChIP-seq
☒	☐ Flow cytometry
☒	☐ MRI-based neuroimaging

Antibodies

Antibodies used	Primary antibodies:<br>rabbit polyclonal anti-TDP-43 antibody (10782-2-AP, 1:500, Proteintech)<br>mouse monoclonal anti-CaMK IV antibody (sc-55501, 1:1,000, Santa Cruz)<br>mouse monoclonal anti-GFP antibody (sc-9996, 1:1,000, Santa Cruz)<br>mouse monoclonal anti-GAPDH antibody (MAB374, 1:2,000, Millipore)<br>mouse monoclonal anti-Neu-N antibody (MAB377, 1:500, Millipore)<br>goat polyclonal anti-Choline Acetyltransferase (ChAT) antibody (AB144P, 1:300, Millipore)<br>rabbit polyclonal anti-Iba1 antibody (019-19741, 1:1,000, Wako Chemicals)<br>mouse monoclonal anti-GFAP antibody (MAB360, 1:2,000, Millipore)<br>rabbit polyclonal anti-calbindin antibody (13176, 1:1,000, Cell Signaling Technology)

rabbit polyclonal anti-PR antibody (23979-1-AP, 1:300, proteintech)
rabbit polyclonal anti-Cre recombinase antibody (15036, 1:500, Cell Signaling Technology)
rabbit polyclonal anti-nucleolin antibody (ab129200, 1:1000, abcam)

Secondary antibodies:
Donkey anti-mouse Alexa Fluor 488 (A21202, 1:300, ThermoFisher SCIENTIFIC)
Goat anti-rabbit Alexa Fluor 594 (111-585-003; 1:300; Jackson ImmunoResearch Laboratories)
Donkey anti-mouse Alexa Fluor 594 (715-585-150, 1:300, Jackson ImmunoResearch Laboratories)
Donkey Anti-Goat Alexa Fluor 568 (ab175704, 1:300, abcam)
HRP-conjugated goat anti-rabbit IgG (111-035-045, 1:10,000, Jackson ImmunoResearch Laboratories)
HRP-conjugated goat anti-mouse IgG (115-035-062, 1:10,000, Jackson ImmunoResearch Laboratories)

Validation

The antibodies were validated by manufactures.

Animals and other organisms

Policy information about studies involving animals; ARRIVE guidelines recommended for reporting animal research

Laboratory animals

Postnatal day 11 - 33, male, C57BL/6J, body weight
5 and 6 months old, male, C57BL/6J, RNA sequencing
2, 3, 6, 10 and 12-16 months old, male/female, C57BL/6J, behavioral test
2, 6 and 12 months old, male/female, C57BL/6J, body weight
2, 6 and 12 months old, male, C57BL/6J, immunohistochemical analysis

Wild animals

No wild animal was used.

Field-collected samples

The study did not involve samples collected from the field.

Ethics oversight

Mice were handled according to a protocol approved by the Soochow University Institutional Animal Care and Use Committee.

Note that full information on the approval of the study protocol must also be provided in the manuscript.
